# The relationship between Hippocampal asymmetry and working memory processing in combat-related PTSD – a monozygotic twin study

**DOI:** 10.1186/2045-5380-2-21

**Published:** 2012-12-01

**Authors:** Timothy Hall, Cherrie Galletly, C Richard Clark, Melinda Veltmeyer, Linda J Metzger, Mark W Gilbertson, Scott P Orr, Roger K Pitman, Alexander McFarlane

**Affiliations:** 1 Discipline of Psychiatry, School of Medicine, University of Adelaide, Adelaide, Australia; 2 Ramsay Health Care (SA) Mental Health Services, Adelaide, South Australia; 3 Cognitive Neuroscience Laboratory and School of Psychology, Flinders University of South Australia, Adelaide, Australia; 4 Veterans Affairs Medical Centre Research Service, Manchester, New Hampshire, England; 5 Massachusetts General Hospital and Harvard Medical School, Boston, USA; 6 The Centre of Military and Veterans’ Health, Adelaide, Australia; 7 Northern Mental Health, Adelaide Metro Mental Health Directorate, Adelaide, South Australia; 8 Harvard Medical School, Boston, USA

## Abstract

**Background:**

PTSD is associated with reduction in hippocampal volume and abnormalities in hippocampal function. Hippocampal asymmetry has received less attention, but potentially could indicate lateralised differences in vulnerability to trauma. The P300 event-related potential component reflects the immediate processing of significant environmental stimuli and has generators in several brain regions including the hippocampus. P300 amplitude is generally reduced in people with PTSD.

**Methods:**

Our study examined hippocampal volume asymmetry and the relationship between hippocampal asymmetry and P300 amplitude in male monozygotic twins discordant for Vietnam combat exposure. Lateralised hippocampal volume and P300 data were obtained from 70 male participants, of whom 12 had PTSD. We were able to compare (1) combat veterans with current PTSD; (2) their non-combat-exposed co-twins; (3) combat veterans without current PTSD and (4) their non-combat-exposed co-twins.

**Results:**

There were no significant differences between groups in hippocampal asymmetry. There were no group differences in performance of an auditory oddball target detection task or in P300 amplitude. There was a significant positive correlation between P300 amplitude and the magnitude of hippocampal asymmetry in participants with PTSD.

**Conclusions:**

These findings suggest that greater hippocampal asymmetry in PTSD is associated with a need to allocate more attentional resources when processing significant environmental stimuli.

## Background

Post Traumatic Stress Disorder (PTSD) is characterised by recurrent and intrusive memories, avoidance, and hyperarousal. PTSD is also associated with impairment in attention, and some studies have shown deficits in learning and memory [1] It has been suggested that disturbed processing of new environmental information in PTSD is due to continuous background re-processing of traumatic events, resulting in modifications of neural circuits [2,3]. The resources required to maintain these autonomous loops may therefore not be available for general attention and memory function. The capacity of working memory has been shown to be directly related to the severity of intrusive memories [4], providing empirical support for this theory.

The hippocampus (HC), located within the ventral medial temporal lobe, is central to the encoding and reconstruction of episodic memories [5]. The hippocampus is part of a large extended network involving the learning of material available within working memory. Plasticity in the physical connections between the HC and the neocortex is crucial for integrating multi-modal sensory information and modifying memory dynamics over time [6]. In addition the HC has a significant role in executive processing systems [7,8]. The HC also contributes to regulation of the hypothalamic-pituitary-adrenocortical system, which is crucial in both acute and chronic responses to stress [9].

The hippocampus, located within the ventral medial temporal lobe, has long been known to be central to explicit long-term recognition memory. Olsen et al. (2012) [10], reviewed recent research showing that the hippocampus is also involved in short-delay recognition and perception. Olsen et al. (2012) [10] concluded that the hippocampus rapidly and continuously forms associations between disparate environmental inputs, including comparing current perceptual input with internal representations. The hippocampus is therefore part of a large extended network involving the recognition and learning of material available within working memory. This network incorporates multiple brain regions, including the prefrontal cortex. Failure in a significant part of this extended network will compromise the functioning of the network as a whole. The hippocampus also contributes to regulation of the hypothalamic-pituitary-adrenocortical system, which is crucial in both acute and chronic responses to stress [9].

Psychological trauma has been found to have damaging effects on the HC [11-14], and animal studies have shown that stress induces changes in hippocampal morphology and function [15,16]. In normal subjects the volume of the right hippocampus is larger than the left hippocampus [17,18]. Numerous studies have shown that hippocampal volume (HCV) is smaller in people with PTSD compared to controls [19-21]. A recent meta-analysis of 39 studies reporting hippocampal volume in PTSD [22] found that people with PTSD and trauma-exposed people without PTSD had smaller left, right and total hippocampal volumes than people never exposed to trauma. This suggests that trauma has an impact on the hippocampus bilaterally. In addition, people with PTSD had smaller volumes of the right, but not the left, hippocampus compared to people who had been exposed to trauma but did not have PTSD. The reduction in right hippocampal volume in PTSD reported by Woon et al. [22] is consistent with an MRI study showing reduced neuronal density in the right medial temporal cortex in people with PTSD, compared to trauma-exposed people without PTSD [23]. These findings suggest that the right hippocampus is differentially reduced in volume in people who develop PTSD.

Disruption of the normal pattern of hippocampal asymmetry has been found in people with schizophrenia [24,25] and in young people meeting criteria for an at-risk mental state for psychosis [26]. Studies in mild cognitive impairment and dementia suggest that there may be changes in hippocampal asymmetry as memory deteriorates [27]. Taken together, these findings demonstrate that abnormal hippocampal asymmetry is found in people subject to pathological processes that affect brain function and manifest as disorders affecting mental state and cognitive function.

Our participants consisted of male Vietnam combat veterans with and without PTSD, and their non-combat exposed monozygotic twins. The contributions of familial vulnerability and trauma exposure to biological abnormalities in this study population have been investigated by Pitman and colleagues [28]. Gilbertson et al. [29] demonstrated that total hippocampal volumes were about 10% smaller in men with severe PTSD, compared to men who did not have PTSD. Consistent with this finding, there was a significant correlation between total hippocampal volume and PTSD symptom severity in the PTSD group. However, these investigators also found that there was no difference in hippocampal volume between participants with severe PTSD, and their combat-unexposed identical twins, suggesting that smaller hippocampal volume might be a biological risk factor, rather than a consequence of trauma exposure and the pathophysiological processes associated with PTSD.

In the present study, we investigated the relationship between hippocampal asymmetry and the capacity to process information about the immediate environment in a sample of identical twins discordant for Vietnam combat exposure. We utilised the amplitude of the event related potential (ERP) P300 component as a measure of the controlled allocation of attentional resources to an anticipated stimulus. The P300 reflects processing of information about a significant stimulus, including context updating, context disclosure, event-categorisation and memory updating [30,31]. The P300 is most commonly elicited by asking participants to identify target tones in an auditory oddball task. The P300 has generators in a number of brain regions, including the temporo-parietal cortex, the cingulate cortex, the thalamus and the inferior and middle frontal cortex [32]. There are two separate generators in the hippocampus, located in the anterior subiculum and the posterior hippocampal body [33].

Numerous studies [34-39] including a meta-analysis by Karl et al. [40] have indicated that the amplitude of the P300 at midline electrodes in relation to trauma-neutral target tones is generally reduced in patients with PTSD. In a study involving the same monozygotic twin population as the present study, Metzger et al. [41] found smaller P300 amplitude in non-medicated, non-smoking twins with PTSD, compared to their combat-unexposed co-twins. In the full sample, there was no difference in reaction time or P300 amplitude between any of the groups (combat veterans with and without PTSD, and their combat-unexposed co-twins respectively). A recent study [42] found that P300 current source density was significantly reduced in people with PTSD in the inferior frontal gyrus, insula, and anterior cingulate.

The aim of the present study was to investigate the relationship between P300 amplitude and hippocampal asymmetry in the twin sample described above. As noted above, it has been established that hippocampal volume has a role in predisposing to PTSD, and there may be further changes related to both trauma exposure and to the development of PTSD. The amplitude of the P300 to trauma-neutral stimuli is generally reduced in PTSD, indicating that the information processing abnormalities characteristic of PTSD are reflected in changes in the P300. In addition, hippocampal generators contribute to P300 amplitude.

Given that both reduced P300 amplitude and greater hippocampal asymmetry (with relatively great volume reduction of the right hippocampus) are likely to reflect pathological changes related to PTSD, we hypothesised that there would be a negative correlation between P300 amplitude to trauma-neutral target tones and the magnitude of hippocampal asymmetry in people with combat-related PTSD (such that smaller P300 amplitude would be found in participants with greater hippocampal asymmetry).

## Methods

### Participants

Participant recruitment, informed consent, and data collection procedures have been described in previous publications [29,43]. Participants completed the 18-item Combat Severity Scale [44], employed in Vietnam Era Twin Registry research and previously validated against combat related medals, which yielded a measure of the severity of their combat exposure; and the Mississippi Scale for Combat-Related PTSD [45], a 35-item instrument for quantifying PTSD and related symptoms. The presence of PTSD in participants was determined by The Clinician-Administered PTSD Scale (CAPS): Current and Lifetime Diagnosis Version [46,47]. All subjects with PTSD included in this study had CAPS scores greater than 65 indicating severe PTSD. In addition, a Structured Clinical Interview for DSM-IV [48] was used to screen for the presence of Axis 1 mental disorders.

Sixty-four male subjects, comprising 32 monozygotic twin pairs where one twin had experienced combat in Vietnam while the other had not, participated in the present study. The mean age of the twin pairs in which the combat-exposed member had PTSD was 53.1 years (SD = 3.3) and the mean age of twin pairs in which the combat-exposed member did not have PTSD was 51.8 years (SD = 2.3) (*F*(1,76) = 3.8, *p* = .050). The mean years of education was 13.5 (SD = 2.6) for combat-exposed subjects with PTSD, 14.3 (SD = 2.8) for their non-combat exposed co-twins, 14.7 (SD = 2.4) for combat-exposed subjects without PTSD, and 14.7 (SD = 2.6) for their non-combat exposed co-twins (NS). Combat-exposed subjects with PTSD had higher combat severity scores (Mean = 7.9, SD = 1.9) than combat-exposed subjects without PTSD (Mean = 3.5, SD = 2.6) and this difference was significant (*F*(1,76) = 67.4, *p* < .001). The participants have been described in more detail by Gilbertson et al. [29].

### Oddball paradigm

Participants were seated upright in a sound-attenuated room. E-A-RTONE (Aearo Company, Santa Barbara, California, USA) earphones were used to present tones binaurally. A five dB descending and ascending staircase method was used to gauge the hearing threshold for a 1,000 Hz test-tone in each participant. All participants completed an auditory target detection task involving 285 tone-stimulus presentations. Three distinctly pitched tones were used: a target (2,000 Hz), a common (1,000 Hz) and infrequent distractor (500 Hz). The entire sequence included 40 target, 40 distracter and 205 common tones with a random inter-stimulus interval varying between 1,950 and 2,050 ms. The task was to identify the high pitched tones as quickly and accurately as possible, by pressing a button with the dominant hand. The presentation sequence was pseudo-randomised to prevent the consecutive occurrence of two infrequent tones. Tones were generated by STIM software (Neuro Scan Inc, Herndon, Virginia, USA) and tone amplitude was verified using an Abbeon AB-85 sound meter (Abbeon, Indianapolis, Indiana, USA).

### EEG measurement

EEG activity was recorded from parietal sites (Pz, P3, and P4) [49], using tin electrodes embedded in a nylon cap (Electro-Cap International, Eaton, UK). EEG was grounded from the forehead and referenced to linked ear lobes. Electrooculogram (EOG) activity was recorded at the outer canthus and infraorbitally to the left eye. Trials with excessive eye-movement artefact (EOG range ±85 μV) were excluded from the averaging process.

EEG impedances were approximately equal and kept below 5 k Ohms. Signals were amplified using Coulbourn High Gain Bioamplifiers (Coulbourn, Allentown, Pennsylvania, USA), band-pass filtered (0.1-150 Hz) and digitally sampled at 1,000 Hz, with a resultant signal sensitivity of .049 μV/bit. The EEG epoch extended from 100 ms pre-stimulus to 900 ms post-stimulus, and was averaged at each site according to stimulus type. It was digitally bandpass filtered between .1 and 14 Hz (12 dB/Oct). Peak measures for P300 were determined from each subject’s averaged waveforms for each stimulus type, using a Neuro Scan interactive scoring program. P300 was defined as the most positive peak between 300–500 ms post-stimulus onset.

### MRI

A 1.5 Tesla MRI scanner (General Electric Signa System, Milwaukee, Wisconsin, USA) was used to perform a whole-brain scan. Automated step algorithms were used to calculate whole brain volume. Subsequently, hippocampal volume was determined by a person blind to the individual’s group characteristics, using a manual tracing procedure [50]. Although hippocampal volume was the focus of the present study, left and right amygdala volumes, and total brain volume, were also measured to provide control data [29]. The reliability of the volume measures was confirmed by a second ‘blind’ rater as previously described [29].

### Statistical analysis

Participants were classified into four groups designated by PTSD diagnosis and combat exposure: (1) combat veterans with current PTSD and (2) their non-combat-exposed co-twins; (3) combat veterans without current PTSD and (4) their non-combat-exposed co-twins. An absolute magnitude HCV asymmetry (HCVA) measure was created for each participant according to the formula: | (right HCV – left HCV)/(right HCV + left HCV) |.

Hippocampal volumes and P300 amplitudes at all electrode sites were screened for outliers more than three standard deviations from the mean. Square root or logarithmic transformations were used as required to correct violations of homogeneity and the Greenhouse-Geisser epsilon correction was used for violations of sphericity. A one-way Analysis of Variance (ANOVA) was used to compare accuracy of target detection between groups, and a repeated measure ANOVA was used to compare P3 amplitude across the three electrodes.

One-way ANOVA was used to compare the total hippocampal volume, and the volumes of the left and right hippocampus, between the four groups. A one-way ANOVA was also performed to compare the asymmetry measures across groups. As EEG scalp potentials are not independent, a principal components analysis was performed across the P300 amplitude data to quantify the number of independent dimensions accounting for 95% of the data variance. A Pearson product–moment correlation coefficient was used to investigate the relationship between the magnitude of asymmetry and P300 amplitude at the three parietal sites (Pz, P3 and P4) in all four diagnosis and exposure groups separately. Finally, separate correlations were performed to evaluate whether significant correlations could be accounted for either ‘right side greater than left’ (R > L) or ‘left side greater than right’ (L > R) hippocampal volume changes. The Pearson product–moment correlation coefficient was also used to investigate the relationships between HC volume and HC asymmetry, total CAPS scores, and scores on individual CAPS dimensions.

## Results

### HC asymmetry

Hippocampal volumes are presented in Table 1. There was a significant main effect for diagnosis for right hippocampal volume (*F*(1,66) = 9.63, *p* = .003) and total hippocampal volume (*F*(1,66) = 8.73, *p* = .004). The mean volume of the left hippocampus was also smaller in the PTSD group but this difference did not reach significance (*F*(1,66) = 3.35, *p* = 0.07). We found that seven subjects with PTSD had a reversal of normal asymmetry with a larger left than right hippocampus, and five had a normal asymmetry with a larger right hippocampus. However, when one-way ANOVAs were performed to investigate between group differences in absolute asymmetry, no significant differences were found between any of the four diagnosis/exposure groups. There was no significant difference between groups in the volumes of the left or right amygdala or in total brain volume.

**Table 1:** Mean (SD) Hippocampal volumes in monozygotic twins with PTSD, their trauma-unexposed twins, and in trauma-exposed and trauma-unexposed monozygotic twins without PTSD

	**PTSD (n = 24)**		**Non-PTSD (n = 46)**	
	**Trauma exposed (n = 12)**	**Trauma unexposed (n = 12)**	**Trauma exposed (n = 23)**	**Trauma unexposed (n = 23)**
Right HC volume (ml)	3.32 (0.59)	3.26 (0.39)	3.76 (0.54)	3.61 (0.48)
Left HC volume (ml)	3.34 (0.46)	3.49 (0.57)	3.65 (0.50)	3.63 (0.45)
Total HC volume (ml)	6.66 (0.83)	6.75 (0.90)	7.41 (0.93)	7.25 (0.69)

### Correlations between HC asymmetry and P300 amplitude

There was no significant difference between the four groups in performance of the target detection task or in P3 amplitude. A principal components analysis of the P300 data across the three electrodes demonstrated that two components accounted for >97% of the total variance, so a Bonferroni corrected *p* value of ≤ .025 was taken as significant.

We found positive correlations between hippocampal volume asymmetry and P300 amplitude in participants with PTSD at sites Pz (*r* = .69, *n* = 12, *p* ≤ .025) and P3 (*r* = .64, *n* = 12, *p* ≤ .025), with higher magnitudes of hippocampal volume asymmetry associated with larger P300 values (Table 2). Neither of the two non-combat exposed co-twin groups, nor the non-PTSD combat-exposed group demonstrated significant correlations between these two variables. The inclusion or exclusion of outliers did not significantly affect the results.

**Table 2:** Bivariate correlations: Hippocampal asymmetry v P300 amplitude

	**Pearson correlation**	**Sig. (2-tailed)**
	**Asymmetry magnitude**	
1: PTSD/Exposed Group N = 12		
Pz P300	0.69	0.01**
P3 P300	0.64	0.03**
P4 P300	0.33	0.29
2: Non-PTSD/Non-Exposed Group (co-twins of Group 1) N = 12		
Pz P300	−0.13	0.70
P3 P300	0.34	0.28
P4 P300	0.41	0.18
3: Non-PTSD/Exposed Group N = 23		
Pz P300	0.12	0.63
P3 P300	0.00	0.98
P4 P300	−0.10	0.67
4: Non-PTSD/Non-Exposed Group (co-twins of Group 3) N = 23		
Pz P300	−0.21	0.37
P3 P300	0.01	0.97
P4 P300	0.02	0.95
**p ≤ .025		

Comparing those with normal HC asymmetry and those with abnormal HC asymmetry, there was no significant difference in P300 amplitude at any electrode, and no group difference in asymmetry of amplitude at the individual electrodes.

### HC asymmetry and symptom measures

In the PTSD group, there was no difference in CAPS scores between those with normal vs abnormal HC asymmetry. There was a negative correlation between total HC volume and re-experiencing symptoms ((*r* = −.56, *n* = 13, *p* ≤ .043), and between left HC volume and re-experiencing symptoms ((*r* = −.71, *n* = 13, *p* ≤ .006).

## Discussion

Based on the results of their meta-analysis, Woon et al. [22] conclude that there may be a differential sensitivity to the effects of trauma, with the right hippocampus being more vulnerable than the left hippocampus. It should be noted however that the subjects from the present study were included in this meta-analysis, contributing about 3% of the PTSD subjects and 11% of the non-PTSD subjects. Our findings were consistent with the findings of this meta-analysis in that the PTSD group had a reversal of normal asymmetry; on average the left hippocampus was slightly larger than the right hippocampus. However their co-twins, and the trauma-unexposed subjects, similarly had slightly larger mean left than right hippocampal volumes. These differences were small and none of these comparisons reached significance. Our results do not support previous findings that the right HC is more sensitive to the effects of trauma than the left HC [22]. In our data, the mean volume of the right HC in trauma-exposed co-twins, both with and without PTSD, was larger than the mean right HC volume of the non-trauma exposed co-twins.

Our results did not support the hypothesis that there would be a negative correlation between P300 amplitude and hippocampal asymmetry. We expected that greater asymmetry, taken to indicate greater pathology, would correlate with a smaller allocation of attentional resources to the working memory task. Instead, we found that although there was no significant asymmetry difference between the four groups, there were significant positive correlations between hippocampal asymmetry and P300 amplitude to target tones in participants with PTSD, at midline (Pz) and left (P3) parietal sites.

The lack of correlation between hippocampal asymmetry and P300 amplitude in the monozygotic co-twins of the PTSD participants, and in the other two groups (including participants with combat exposure) suggests that the correlations are associated with PTSD itself, rather than being genetically determined or related to combat exposure. It has previously been suggested [51] that the presence of such correlations in subjects with psychiatric illness, but not in normal subjects, may reflect disease-associated pathology. This conclusion might be strengthened if there was a correlation between P300 amplitude or the magnitude of hippocampal asymmetry, and symptom severity. Bae et al. (2011) [42] have demonstrated associations between P300 current source density (CSD) in regions in the frontal, parietal and temporal cortices, and various PTSD symptoms including re-experiencing and increased arousal (positively correlated with P300 CSD) and avoidance and numbing (negatively correlated with P300 CSD). Investigation of the associations between hippocampal volume asymmetry, P300 amplitude, and symptom severity, was beyond the scope of the present study. However, further research is required looking at the links between brain structure, brain function, and measures of disease severity. In addition, we did not have information about handedness, although co-twins would be expected to be matched for handedness. The proportion of people with mixed or right hemisphere dominance in the general population is small so it is unlikely that the presence of such subjects would have affected the overall conclusions.

Whilst most ERP studies of subjects with PTSD have found P300 amplitude reduction, an increase in P300 amplitude to trauma-neutral stimuli has been reported in a population of nurses who developed PTSD following exposure to wounded combatants in Vietnam [52]. P300 amplitude has been shown to be increased to trauma-relevant stimuli in people with PTSD, indicating an increase in allocation of attention resources towards elements in the environment that may be immediately threatening [43].

It is possible that the larger P300 in our subjects with greater hippocampal asymmetry might reflect a greater allocation of attention, in an effort to overcome difficulties performing the task. Amman et al. [53] report that in multiple sclerosis, a disease that is associated with neuropsychological impairment, patients showed stronger fMRI activation change in the right parahippocampal cortex and in the middle and medial frontal regions during the performance of simple tasks involving attention and working memory, compared to normal controls. There was a linear increase in activation with increasing task complexity, until the task load became excessive. Similarly, Fabiani and Friedman [54] found increased P300 amplitude was associated with poorer performance on the Wisconsin Card Sorting Test.

Our results might therefore indicate that the subjects with PTSD needed to give greater attention to the task in order to achieve normal results. Both auditory and visual stimulus processing in working memory are associated with hippocampal activation [55], and the hippocampus is involved in the processing mechanisms that determine attentional allocation and P300 amplitude [56]. A large asymmetry of hippocampal structures may necessitate a change in the neurophysiology of non-traumatic stimulus encoding, reflected in the enlarged P300 components.

Working memory capability has been shown to be decreased in those individuals who experience intrusive memories, indicating that a proportion of finite working memory resources is required during re-experiencing of traumatic memories, leaving fewer resources available for updating online content [57]. Nadel et al. [5] have shown that the hippocampus is intimately related to the processing and retrieval of spatial and episodic memories, long after they have been established, supporting the role of the hippocampus in the re-experiencing of intrusive memories. Our PTSD subjects may therefore have less capacity to process non-trauma related stimuli, because of the ongoing processing of intrusive trauma-related material. As noted above, this could result in a greater allocation of effort, to overcome these difficulties. Our results are consistent with the proposition that the increased allocation of attention was maximal in those with greatest hippocampal pathology, reflected in greater hippocampal asymmetry. In PTSD, the availability of hippocampal neurons required for encoding the stimuli in the oddball task may act as a rate-limiting step in non-trauma related information processing. In addition, the correlation between smaller HC volume and re-experiencing symptoms could suggest that HC damage is associated with an inability to process and eventually recover from traumatic memories.

Whilst this study focussed on hippocampal pathology, following on from previous research demonstrating hippocampal abnormalities associated both with trauma, and with PTSD, there is also evidence of volume reductions in other brain regions in PTSD. Araki et al. (2004) [58] investigated the relationship between lower P300 amplitude and anterior cingulate grey matter volume in people who developed PTSD as a result of the Tokyo subway sarin attack. The numbers were quite small (8 subjects with PTSD and 13 subjects without PTSD who had been exposed to the sarin attack). There was a trend-level correlation (p = 0.077) between P300 amplitude at Pz and left anterior cingulate grey matter volume. Further research into brain structure and function in PTSD should therefore extend to other brain regions beyond the hippocampus.

## Conclusions

In conclusion, the hippocampus is clearly sensitive to the effects of trauma, with changes in both structure and function. Further, smaller hippocampal volume appears to be associated with vulnerability to developing PTSD when the person is exposed to trauma. Hippocampal volumes and hippocampal asymmetry have therefore been taken as reflecting both genetically determined vulnerability and trauma-related damage to the hippocampus. Our monozygotic twin sample enables us to control for genetic factors, so hippocampal volume differences are therefore related to trauma exposure, in some cases with the addition of the pathological processes associated with PTSD. The oddball P300 event related potential reflects the allocation of attentional resources during working memory processing, and hippocampal structures make a substantial contribution to P300 amplitude. Previous research has shown that the P300 is sensitive to the effects of PTSD [38]. Our results show that, in people with PTSD only, there is a positive correlation between hippocampal asymmetry and allocation of attentional resources. This could be because a proportion of finite processing capacity is being utilised by the constant re-experiencing of traumatic memories, leaving limited resources for non-trauma related cognitive tasks.

## Competing interests

The authors declare that they have no competing interests.

## Authors’ contributions

TH undertook the analyses of hippocampal asymmetry and ERP measures. CG, CRC and AM were responsible for the planning and supervision of this work. LM, MV, CRC, AM, RP and SO undertook the initial ERP analyses. MG, RP and SO undertook the measurement of hippocampal volume. TH, CG, CRC and AM wrote the first drafts of the manuscript. All authors read and approved the final manuscript
